# Amyloid β-based therapy for Alzheimer’s disease: challenges, successes and future

**DOI:** 10.1038/s41392-023-01484-7

**Published:** 2023-06-30

**Authors:** Yun Zhang, Huaqiu Chen, Ran Li, Keenan Sterling, Weihong Song

**Affiliations:** 1 National Clinical Research Center for Geriatric Disorders, Xuanwu Hospital, Capital Medical University, Beijing, China; 2 The Second Affiliated Hospital and Yuying Children’s Hospital, Institute of Aging, Key Laboratory of Alzheimer’s Disease of Zhejiang Province, Wenzhou Medical University, Wenzhou, Zhejiang China; 3 Townsend Family Laboratories, Department of Psychiatry, The University of British Columbia, Vancouver, BC V6T 1Z3 Canada; 4 Oujiang Laboratory (Zhejiang Lab for Regenerative Medicine, Vision and Brain Health), Wenzhou, Zhejiang China

**Keywords:** Neurological disorders, Drug discovery

## Abstract

Amyloid β protein (Aβ) is the main component of neuritic plaques in Alzheimer’s disease (AD), and its accumulation has been considered as the molecular driver of Alzheimer’s pathogenesis and progression. Aβ has been the prime target for the development of AD therapy. However, the repeated failures of Aβ-targeted clinical trials have cast considerable doubt on the amyloid cascade hypothesis and whether the development of Alzheimer’s drug has followed the correct course. However, the recent successes of Aβ targeted trials have assuaged those doubts. In this review, we discussed the evolution of the amyloid cascade hypothesis over the last 30 years and summarized its application in Alzheimer’s diagnosis and modification. In particular, we extensively discussed the pitfalls, promises and important unanswered questions regarding the current anti-Aβ therapy, as well as strategies for further study and development of more feasible Aβ-targeted approaches in the optimization of AD prevention and treatment.

## Introduction

Alzheimer’s disease (AD) is the most common neurodegenerative disorder leading to progressive cognitive decline with pathological hallmarks of senile plaque and neurofibrillary tangle formation in the brain. In 1984, Glenner & Wong discovered that the amyloid β protein (Aβ) is the central component of extracellular amyloid plaques in AD.^1^ Since then, Aβ has been considered as a driver of Alzheimer’s pathological processes and the “amyloid cascade hypothesis” has become a leading theory of AD pathogenesis.^2^ Over the past decades, targeting Aβ has been the main direction of developing AD treatment.^3–6^ However, the repetitive failures of Aβ-targeted clinical trials have cast considerable doubt on this hypothesis. Anti-Aβ therapy has now become a significant controversy in AD drug development and treatment.

**Fig. 1:**
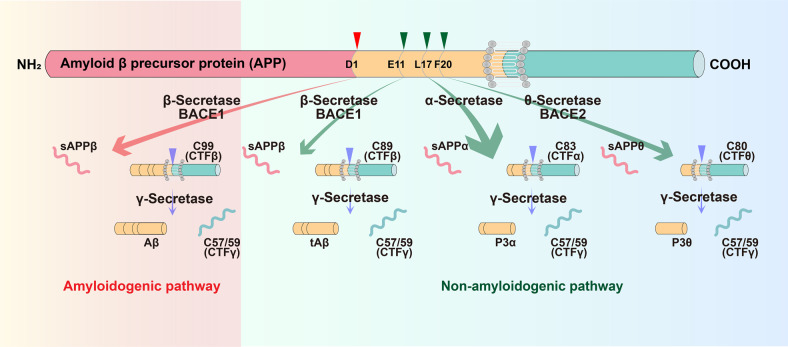
Amyloidogenic and non-amyloidogenic processing pathways of APP. In the amyloidogenic pathway, BACE1 first cleaves APP at the Asp^1^ site to generate sAPPβ and a 99-amino acid membrane-bound C-terminal fragment (CTF) C99. Subsequently, γ-secretase cleaves C99 to release Aβ and CTFγ. Under physiological conditions (non-amyloidogenic pathways), APP is mostly cleaved first by α-secretase within Aβ domain at the Aβ Leu^17^ site, generating a secreted form of APP (sAPPα) and an 83-amino acid membrane-bound C-terminal fragment (CTF) C83, thus precluding Aβ production; BACE1 predominantly processes APP at the Aβ Glu^11^ β-secretase site to generate C89, and γ-secretase cleaves C89 to produce a truncated Aβ_11-40_; BACE2 cleaves APP at the Aβ Phe^20^ θ-secretase site to generate C80 and precludes Aβ generation. APP amyloid precursor protein, BACE1 β-site APP-cleaving enzyme 1, sAPP secreted APP, CTF C-terminal fragment, Aβ amyloid-β, tAβ truncated amyloid-β, BACE2 β-site APP-cleaving enzyme 2

Aβ is generated from the amyloid precursor protein (APP) by sequential cleavage of β- and γ-secretase. However, the non-amyloidogenic pathway is the predominant pathway in vivo.^7^ APP is mostly cleaved first by α-secretase within Aβ domain at the Aβ Leu^17^ site in the non-amyloidogenic pathway, generating a secreted form of APP (sAPPα) and an 83-amino acid membrane-bound C-terminal fragment (CTF) C83, thus precluding Aβ production. The beta site APP cleaving enzyme 1 (BACE1), the β-secretase, and its homolog BACE2, the θ-secretase, also contribute to the non-amyloidogenic pathway.^7,8^ Under physiological conditions, BACE1 predominantly processes APP at the Aβ Glu^11^ β-secretase site to generate C89, and γ-secretase cleaves C89 to produce a truncated Aβ_11-40_.^7,8^ BACE2 cleaves APP at the Aβ Phe^20^ θ-secretase site to generate C80 and precludes Aβ generation.^9–11^ Two enzymatic cleavages of APP by BACE1 and γ-secretase are required to produce Aβ in the amyloidogenic pathway. BACE1 first cleaves APP at the Asp^1^ site to generate sAPPβ and C99. Subsequently, γ-secretase cleaves C99 to release Aβ and CTFγ. γ-secretase is a presenilins 1 (PS1)-containing macromolecular complex^12–16^ and this high molecular weight complex also requires nicastrin, anterior pharynx-defective 1, and PEN-2 for its enzymatic activity^17,18^ (Fig. 1).

The balance between continual Aβ generation and efficient clearance is important for Aβ homeostasis to prevent its toxic aggregation into misfolded assemblies.^19^ Similar to other brain metabolites, Aβ clearance depends on different pathways including enzyme degradation, crossing the blood–brain barrier (BBB), interstitial fluid (ISF) bulk-flow and CSF absorption.^19,20^ The BBB is composed of endothelial cells connected by tight junctions to form a selectively permeable system.^21^ The transport of soluble Aβ across brain endothelial cells to the peripheral circulation is mainly via low density lipoprotein receptor-related protein 1(LRP-1) and ABC transporter sub-family A and B member 1 (ABCA1 and ABCB1),^22,23^ while receptors for advanced glycosylation end-products (RAGE) is responsible for circulating Aβ entering into the brain.^24^ It has been identified that the expressions of the two blood efflux transporters LRP1 and ABCB1 were reduced during AD, whereas the expression of the blood influx transporter RAGE is elevated.^21,25^ The perivascular drainage pathway plays a vital role in ISF bulk-flow clearance of Aβ.^26^ Failure of perivascular drainage of Aβ altered Aβ homeostasis associated with synaptic dysfunction and cognitive impairment, leading to the development of AD.^27^ CSF absorption clearance of Aβ depends on factors including CSF production by the choroid plexus, integrity of the blood-CSF barrier, relevant transporters and CSF lymphatic absorption.^28^ In AD, the structural integrity of the blood-CSF barrier is destroyed, resulting in aberrant Aβ clearance.^29^ Enzymatic pathways for Aβ degradation include the zinc metalloendopeptidases, insulin-degrading enzyme (IDE), matrix metalloproteinase (MMPs), angiotensin converting enzyme (ACE), and endothelin-converting enzyme (ECE), serine proteases, cystein proteases, and kallikrein-related peptidase 7.^30,31^ In the hippocampus of AD patients, the enzymes IDE, ACE and NEP had decreased activity.^30^ AD model mice also showed the impaired Aβ degradation system.^21,32^ In GWAS, many genetic risk factors for AD (e.g. *RIN3*, *CLU* and *PTK2B*) are linked to Aβ degradation.^33,34^

Extensive genetic studies have supported the causative role of Aβ accumulation in AD pathogenesis. Down syndrome (DS) patients with trisomy-21 having extra copy of *APP* gene develop typical Alzheimer’s neuropathology including amyloid plaques and neurofibrillary tangles.^35–37^ Mutations in *APP, presenilin 1* (*PSEN1)* and *PSEN2* genes that increase Aβ production, elevate Aβ_42_/Aβ_40_ ratio and promote plaque formation cause autosomal dominant early-onset familial AD (FAD), implicating a role of altering APP processing in AD pathogenesis.^7,38,39^ In contrast, an *APP* mutation identified in the Icelandic population reduces Aβ production, leading to protection against cognitive decline in the elderly.^40^ Both genetic (e.g., *ApoE4, TREM2*) and non-genetic (e.g. diabetes, obesity, stroke, or physical inactivity) risk factors for late-onset sporadic Alzheimer’s disease (SAD) have also been identified to increase Aβ generation and/or reduce Aβ clearance for its accumulation.^4,41–45^ These studies suggest that Aβ accumulation drives disease progression in both FAD and SAD and thus illustrates why clinical trials involving anti-Aβ therapies have garnered so much attention in the Alzheimer’s community.

Recently, Aβ-based therapy has received encouraging results. Aducanumab, a monoclonal antibody against Aβ aggregates, has obtained the FDA’s approval as an Alzheimer’s drug for its ability to reduce the level of Aβ plaques in patients with early AD or mild cognitive impairment (MCI).^46–48^ On Nov 30 2022, Eli Lilly and Company (https://investor.lilly.com/news-releases/news-release-details/lilly-shares-positive-donanemab-data-first-active-comparator) announced the result of the first active comparator study (TRAILBLAZER-ALZ 4), which showed that donanemab, another monoclonal antibody targeting deposited plaques had outperformed aducanumab-avwa treatment in terms of brain amyloid clearance in patients with early symptomatic AD.^49^ At the same time, results from the highly anticipated CLARITY AD study were published, showing that 18 months of treatment with lecanemab, a humanized IgG1 monoclonal antibody targeting Aβ soluble protofibrils, reduced markers of amyloid and moderately improved cognitive decline in patients with early AD.^50^ Recently, the FDA approved lecanemab as the second-ever monoclonal antibody to treat AD. ANAVEX®2-73 (Blarcamesine), which targets sigma-1 and M1 muscarinic receptors, has also demonstrated its disease-modifying activity in AD transgenic mice (3xTg-AD), including reducing amyloid and tau pathologies as well as improving cognitive deficits.^51,52^ The results of its Phase 2B/3 study, presented at the Clinical Trials on Alzheimer’s Disease (CTAD) Congress 2022, showed that 48 weeks of blarcamesine treatment significantly reduced cognitive decline in patients with early AD. This series of positive results offers a fresh hope and indicates that Aβ-based therapy may be indeed the right direction to be followed. In this review, we summarized the history and current understanding of the “amyloid cascade hypothesis”. In particular, we discussed the pitfalls, promise and important unanswered questions about the current anti-Aβ therapy, which will provide a foundation for further studying and developing more feasible Aβ-targeted strategies to optimize AD prevention and treatment.

## The history of amyloid cascade hypothesis (Fig. 2)

In 1984, Aβ was identified as the primary component of extracellular amyloid plaques in AD,^1^ which is the unique pathological hallmark of the disease.^53^ Hardy and Higgins then proposed “the amyloid cascade hypothesis” in 1992, positing that Aβ deposits in the brain are the initiating event of AD pathogenesis, resulting in subsequent tau tangle formation, neuronal loss and dysfunction as well as cognitive decline.^2^ Since then, many genetic and non-genetic studies have supported this hypothesis. Down syndrome with *APP* gene triplication or *APP* locus duplications produces an increase in Aβ production and the Aβ_42_/_40_ ratio, leading to plaque formation and cognitive decline. APP mutations increase total Aβ and the ratio of Aβ_42_/Aβ_40_, leading to early-onset Alzheimer’s disease (EOAD). The *apolipoprotein E (APOE)* and *clusterin (CLU)*, the strongest genetic risk factors for late-onset Alzheimer’s disease (LOAD), has also been identified to influence Aβ seeding and clearance.^4,41^

**Fig. 2:**
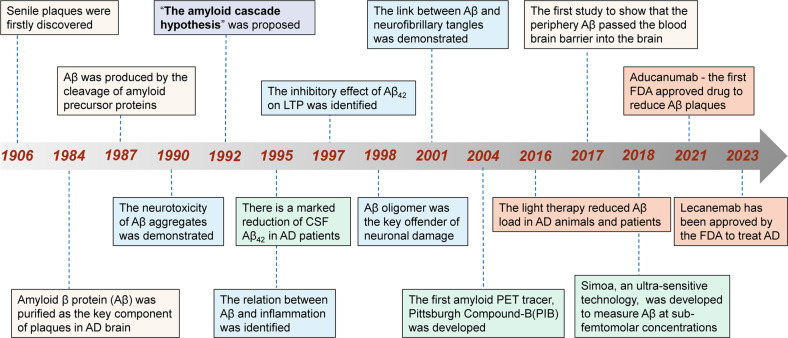
Milestone of the amyloid cascade hypothesis and its applications. Yellow box: key research findings; blue box: the Aβ-related toxicity; green box: the diagnostic application; pink box: important drug and non-drug anti-Aβ therapies. AD Alzheimer’s disease, CSF cerebrospinal fluid, FDA food and Drug Administration, LTP long-term potentiation

### Morphology of Aβ aggregates

After secretion, Aβ first aggregates into different soluble species that then change their conformation into cross-β-sheet fibrils to form plaques. There are two types of amyloid plaques: classical and diffuse ones. The classical plaques have a compact core of Aβ surrounded by an optically clear area and an outer corona.^54^ The corona consists of both neuronal and glial elements, including degenerative neuronal processes (neurites) along with reactive astrocytes and microglia.^55,56^ Diffuse plaques comprise very small, often stellate assemblies scattered about the parenchyma. It refers to the fact that the Aβ accumulation is widely spread or scattered, but not concentrated.^57–59^ Without consideration of the nature of the Aβ deposits (e.g. thread-like or punctate), “diffuse” thus denotes only the characteristics of the Aβ deposits, and not the dysmorphic neuritis or any other component of the plaques. A recent study showed that it is the classical plaques with inflammatory cells rather than diffuse plaques that correlate with the cognitive impairment during AD.^60^

### Pathological role of Aβ aggregates (Fig. 3)

The amyloid cascade hypothesis has been the leading model of AD pathogenesis since it was proposed, and the hypothesis has being revised over time. The original hypothesis focuses on large insoluble Aβ fibrils as the key offender of neuronal damage, while growing evidence supports that the Aβ oligomers exist and exert their neurotoxicity independently of mature fibrils.^61^ The amyloid-β oligomer (AβO) hypothesis suggests that AD pathogenesis was instigated by soluble, ligand-like Aβ oligomers.

**Fig. 3:**
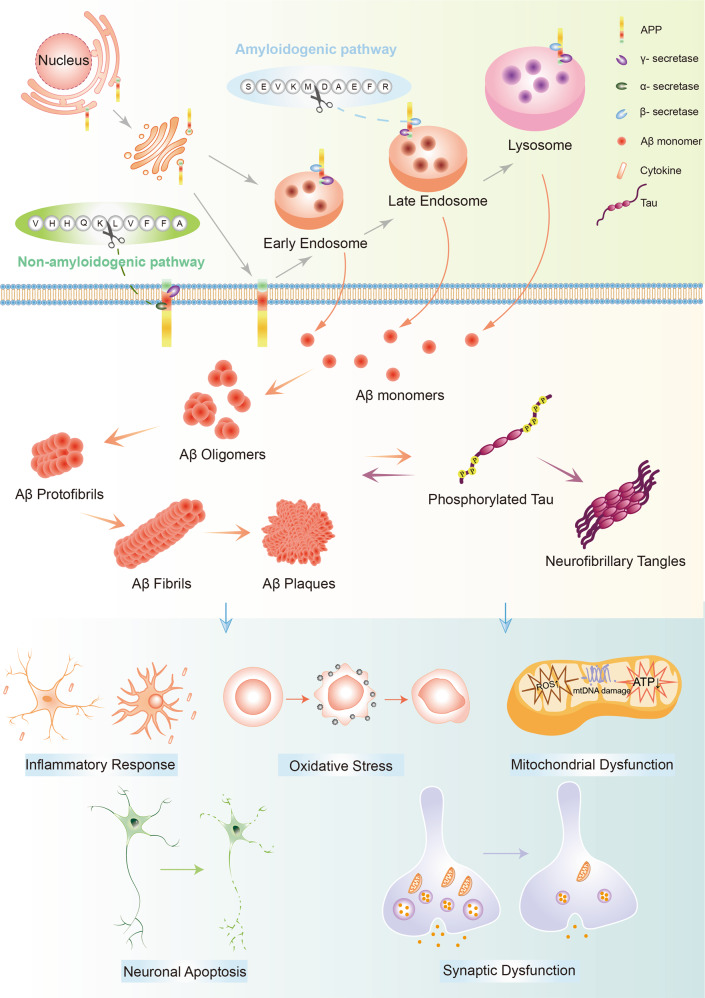
The generation, aggregation and pathological functions of Aβ. Aβ is generated from APP by sequential cleavage of β-secretase (beta-site APP cleaving enzyme 1, BACE1) and γ-secretase. BACE1 first cleaves APP at the Asp^1^ site to generate sAPPβ and C99. Subsequently, γ-secretase cleaves C99 to release Aβ (Aβ_1-40/42_ are the most common isoforms) and CTFγ. After secretion, Aβ peptides first oligomerize into different soluble species then convert their conformation into profibrils and cross-β-sheet fibrils, forming amyloid plaques. Aβ aggregates interact with tau proteins to exert the toxic effects. In addition, they contribute to other AD pathological features including neuroinflammation, oxidative stress and mitochondrial dysfunction, leading to neuronal death and dysfunction. Aβ amyloid β, APP amyloid precursor protein

#### Interact with cell membrane

Aβ aggregates can directly interact with the lipid and cholesterol components of the cell membrane, forming channels and destroying membrane integrity and permeability, which allows Ca^2+^ entering into the cell, leading to LTP inhibition and neuronal death.^62,63^ For example, AβOs bind to sialic acid-containing GM1 ganglioside on cell membrane to induce LTP impairment.^64^ On the other hand, cholesterol-rich lipid rafts provide an optimal environment for Aβ synthesis and enhance the interaction of Aβ with the membrane.^65^ Both β- and γ-secretases show increased enzymatic activity in the lipid rafts with higher cholesterol level, while non-amyloidogenic α-secretase activity is inhibited by cholesterol.^66–69^ In addition, It is well established that cholesteral-containing lipid membrane can influence Aβ seeding and aggregation.^70,71^ As a nucleation process, cholesterol and GM1-rich lipid rafts accelerate Aβ aggregation by binding with Aβ to stabilize its structure.^72,73^ Thus, reduction of cholesterol in endosomes or lysosomes ameliorates Aβ aggregation and its toxicity in mouse models.^74^

#### Interfere with synaptic plasticity

Impaired synaptic function is considered to be an early and key pathology of AD. Synaptic loss is also closely correlated with cognitive decline in Alzheimer’s patients.^75^ Aβ oligomers change the morphology and density of synapses, leading to the impairment of synaptic plasticity.^76,77^ As a glutamate receptor, functional NMDARs regulate the formation of synapes and synaptic plasticity.^78^ AβOs directly disturb the activity of NMDARs and impair NMDAR-mediated signaling pathways (e.g. Wnt/β-catenin signaling pathway), leading to synaptic loss and the reduction of spinal density.^79^ Furthermore, AβOs destroy Glu-recycling at the synapse by increasing glutamate release, reducing glutamate uptake and impairing glutamate transporters, which causes the overactivation of extrasyaptic NMDARs, ultimately leading to LTP suppression, LTD enhancement, and synaptic loss.^80^ α-Amino-3-hydroxy-5-methyl-4-isoxazolepropionic acid receptor (AMPAR) is another glutamate receptor containing four subunits GluA1-4, which makes up to 80% of the excitatory synapses in the CA1 region of hippocampus.^81,82^ Many studies have shown that AMPARs also take part in the modulation of synaptic plasticity.^83,84^ However, AβOs induce AMPAR ubiquitination and degradation, leading to the loss of AMPARs followed by the suppression of synaptic transmission.^85,86^ Recently, two parallel studies have further investigated the underlying mechanism of the Aβ’s detrimental effect over synaptic transmission.^87,88^ They found that intracellular administration of the AβOs rather than administration of the AβOs at the extracellular level altered the synaptic transmission and fast axonal transport via the casein kinase 2 (CK-2) activation. In addition, the LTP inhibtion and LTD enhancement mediated by Aβ aggregates further result in the shrinkage of dendritic spines by remodeling actin.^89,90^ Furthermore, Aβ aggregates and hyperphosphorylated tau protein exert synergistic effect on impairing synapse function.^91–93^ AβOs induce tau hyperphosphorylation and accumulation in dendritic spine, which further lead to synaptic loss and dysfunction.^94,95^ The level of pathological tau in AD patients is correlated with the severity of impaired synaptic plasticity and cognitive dysfunction.^96^ The pathological tau interacts with the presynaptic compartments including synapsin-1, synaptophysin, to inhibit the mobility and release of synaptic vesicles, leading to the development of AD.^97–99^ Missorted tau proteins at postsynaptic terminals interacts with the subunits of AMPARs and NMDARs, leading to the excessive activation of glutamate receptors, Ca^2+^ influx, impaired LTP and enhanced LTD.^92,100,101^ It has been demonstrated that the absence of tau proteins prevent Aβ-induced LTP impairment mouse hippocampal slices.^102^ Another study also indentified that reduction of tau could ameliorate Aβ-induced Ca^2+^ influx into neurons and AD-related excitotoxicity in vivo.^103^ These findings suggest that the synaptic toxicity induced by Aβ was dependent on pathological tau proteins to some extent.

#### Aβ-induced tauopathy

Beside Aβ plaques, neurofibrillary tangles (NFTs) containing hyperphosphorylated tau are also a hallmark of Alzheimer’s pathology.^104–107^ Over past dozen years, a growing number of evidence has indicated the importance of Aβ-tau interaction in Alzheimer’s pathogenesis. In the tripple transgenic mice (3xTg-AD), extracellular Aβ accumulates in the neocortex and hippocampus followed by tau seeding into fibrillar tangles.^108^ Injection of Aβ aggregates into brain of P301L mutant tau transgenic mice triggers a five-fold elevation in NFTs in the amygdala.^109^ In the clinical setting, neuroimaging of sporadic Alzheimer’s patients show the increased cortical tau-PET ligand retention only in the presence of Aβ accumulation, which is also associated with cortical atrophy in AD.^110^ In addition, longitudinal studies idenfied that antecedent Aβ aggregates could successfully predict the subsequent tau changes in the inferior temporal cortex.^111^ As the upstream factor, Aβ triggered the hyperphosphorylation of tau protiens,^112–114^ which synergistically induced neuronal impairment and cognitive deficits.^111,115^ Aβ accelerated the tau hyperphosphorylation by the activation of cyclin-dependent kinase 5 (CDK5) and glycogen synthase kinase 3 (GSK-3).^116–118^ GSK-3β, which is inextricably associated with Aβ production and accumulation,^119^ is a key trigger of tau phosphorylation and aggravates Aβ-induced tau toxicity.^120^ CDK5-P25 phosphorylates tau at sites of Thr181, Ser199, Ser202, Thr205, Thr212, Ser214, Ser217, Thr231, Ser235, Ser396, and Ser404.^121^ Thus, inhibitors of GSK-3β or CDK-5 such as AZD1080 and roscovitine, markedly reduced the levels of tau phosphorylation and prevented further tau aggregation.^122,123^ Further studies have found that mitogen-activated protein kinases (MAPKs) including ERK1/2, SAPKs and p38 are also involved in Aβ-induced formation of PHF-tau during AD.^124,125^ Cellular prion protein (PrPC) has been found as a receptor for toxic Aβ oligomers to induce LTP loss and cognitive impairment in AD model mice.^126,127^ PrPC has been also detected in Aβ plaques in Alzheimer’s patients,^128–130^ which activates Fyn kinase and phosphorylates tau by the GluN2B subunit of NMDARs.^100,131–133^ In addition to its stimulatory effect on tau phosphorylation, Aβ also affected tau oligomerization and tangle formation.^134^ Aβ triggered caspase-3 (CASP3)-induced cleavage of tau at Asp421 to yield an N-terminal product, which self-aggregated and further assembled into neurotoxic oligomers.^134,135^ Tau oligomers not only led to neuronal damage but also bound to astrocytes and microglia to induce neuroinflammation.^136^ In hippocampal neurons, Aβ also induced the activation of calpain-1 and generated a 17-kDa tau fragment, resulting in neurite degeneration and neuronal death.^137^

Meanwhile, the toxic state of tau proteins also influence Aβ production. Thus, knocking out the tau genes in the APP/PS1 mice inhibited the amyloidogenic pathway of APP processing, Aβ production and the amyloid plaque formation.^138^ Furthermore, the neurotoxicity of Aβ is tau-dependent. Absence of tau on NMDARs of spines successfully prevented the toxic effect induced by the binding of Aβ to NMDARs.^92^ A recent study proposed that the phosphorylation of tau at Tyr18 by Fyn kinase also blocked Aβ toxicity.^139,140^ Aβ promoted the phosphorylation and activation of Fyn kinase, which further migrated into dendritic spines, leading to synaptic impariment.^141,142^ Tau protein mediated Aβ toxicity by interacting with Fyn kinase via its amino-terminal projection domain (PD).^143^ Accordingly, inhibition of Fyn improved the cognitive deficits in transgenic mice with Aβ and tau depositions.^144,145^ PET and CSF tests also indicate the synergy between Aβ and tau, which leads to brain dysfunction and cognitive impairment.^146–148^ In contrast, Aβ and tau have antagonistic effects on neural circuit.^149^ Tau induces the profound silencing of circuits by blocking Aβ-dependent hyperactivity in the cortex.^150^

#### Induce inflammation

Neuroinflammation is chronic inflammation in the CNS, which is attributed to activated microglia and astrocytes to produce numerous pro-inflammatory cytokines.^151^ Growing evidence demonstrates that neuroinflammation plays a vital role in the neuropathological changes in AD.^152–154^ In addition, patients who are with long-term nonsteroidal anti-inflammatory drugs (NSAIDs) for treating other diseases such as rheumatoid arthritis, showed a 50% reduction in the risk for developing AD.^155^ It has been reported that the inflammation-associated proteins and cells were localized closely to Aβ plaques in AD brain.^156^ However, the possible underlying mechanisms are still unclear. One potential explanation for the activated glia cells in AD brain could be the response to Aβ produced largely by neurons.^157,158^ Aβ shares structural similarities with antimicrobial peptides (AMPs) and viral fusion domains, which stimulates glia cells to secrete a mass of pro-inflammatory cytokines.^159^ Similar to AMPs, Aβ aggregates can also induce pores in cell membranes, which allow a variety of stimuli to activate glia cells.^160^

Microglia comprise around 10–15% of all glial cells, which are the resident macrophages within the CNS.^161^ In a healthy adult brain, microglia are in a resting state and highly ramified morphology with small somas.^162^ These cells communicate with surrounding environments including neurons, astrocytes and blood vessels to maintain the development and homeostasis of the CNS.^163,164^ When microglia recognize the insults of the CNS, they respond to the injury or invasion by a morphological change, resulting in cell enlargement and migration.^165^ In the development of AD, it has been suggested that Aβ aggregates are the primary driver to activate microglia and set them into motion. Activated microglia migrated to the Aβ deposition and stimulated the phagocytosis of Aβ.^166–168^ Thus, factors such as CD33, which impedes Aβ phagocytosis by microglia, has been considered to increase the risk for suffering from AD.^169^ However, the prolonged activation of microglia become enlarged and are no longer able to exert their phagocytic function. In contrast, their capacity of pro-inflammatory cytokine production is unaffected, contributing to an exacerbation of AD pathology including Aβ accumulation and neuronal damage.^170,171^ To compensate the impaired clearance of Aβ, peripheral macrophages are recruited to the brain in an effort to clear Aβ plaques, which likely worsens the sustained inflammation and thus AD pathologies.^172,173^ Compared with microglia distal to the amyloid in AD brain tissues, there is an increased expression of TREM2 in the cells close to Aβ plaques.^174,175^ Increased TERM2 experession has been found in human AD blood, indicating the important role of peripheral TREM2 in Alzheimer’s pathogenesis.^176,177^ Using flow cytometry identified that these cells also contained high levels of CD45, Ly6c, and CD11b, which highly express in peripheral macrophages as well.^174^ Partial or completed deletion of TREM2 markedly reduced the number of Aβ-associated macrophages and increased cerebral Aβ plaques in AD model mice.^174,175,178^ The reduction of TREM2 in Aβ-associated macrophages also altered astrocytosis detected by glial fibrillary acidic protein (GFAP) and S100β.^178^

As the most abundant glial cells in the CNS, astrocytes play an essential role in the communication with neurons and regulation of synapse formation and function.^179^ Under pathological conditions, astrocytes become reactive, which are characterized by cell hypertrophy with GFAP and vimentin expressions as well as the release of cytotoxins.^180–182^ The reactive astrocytes are close to Aβ plaques in brains from AD patients and rodent models.^183,184^ Astrocytes response to Aβ aggregates in a TLR-dependent manner, which further activates the target genes to produce proinflammatory factors.^185,186^ The excessive production of proinflammatory cytokines such as TNF-α or IFN-γ modulated the APP processing in astrocytes, leading to the increased Aβ levels and toxicity.^187^ These studies have revealed a significant role of reactive astrocytes in the loop between inflammatory cytokines and Aβ load.^188^ Disturbed this cross-talk has been considered to underlay Alzheimer’s pathogenesis. Impaired astrocyte activity also increased the number of microglia surrounding Aβ plaques and altered the microglia status.^189^ In turn, microglia could alter the status of astrocytes. The activated microglia secreted IL-1α, TNF and C1q cytokines to further induce A1 reactive astrocytes, which are neurotoxic and increased in human AD post-mortem tissues.^190^ In addition, Aβ produced by neurons induced the complement protein C3a released by astrocytes via NFκB signaling, which interacted with the receptors (C3aRs) on microglia and neurons to aggravate Aβ aggregate loads and cognitive impairment.^191^

#### Mitochondrial dysfuntion and oxidative stress

Mitochondria are the major powerhouses for cells, where oxidative phosphorylation (OXPHOS) occurs to generate ATP for maintaining the optimal neuronal activities.^192^ Mitochondria are essential for the glutamate synthesis, synaptic transmission and calcium regulation.^193,194^ Disrupted energy metabolism has been found in early AD and precedes the disease development, suggesting the core role of mitochondria dysfunction in Alzheimer’s pathogenesis.^195,196^ Soluble Aβ oligomers disrupted the balance between mitochondrial fission and fusion, leading to significant mitochondrial dysfunction.^197,198^ Excessive mitocondrial fission is a key modulator of Aβ toxicity.^199^ Thus, restoration of mitochondrial fission rescued APP- or Aβ-induced mitochondrial abnormality and neuronal damage.^200,201^ Only 1% of mitochondrial proteins are synthesized in the mitochondria itself. Instead, most proteins of the mitochondria are synthesized by cytosolic ribosomes then imported into the organelle.^202^ APP- or Aβ-induced impairment of mitochondrial import pathway has been considered as a hallmark of AD.^203–205^ It has been demonstrated that APP blocked mitochondrial import machinery and impaired mitochondrial function in AD brain by forming a complex with translocases of the inner and outer mitochondrial membranes.^204^ In addition, endoplasmic reticulum (ER)-mitochondria contact sites provide a platform to regulate important cellular activities, including synthesis of phospholipids, calcium transport between ER and mitochondria, regulation of mitochondrial homeostasis, activation of inflammasome, and induction of apoptosis.^206,207^ Alteration of mitochondria-associated endoplasmic reticulum membrane (MAM) signaling has been implicated in neurodegenerative diseases such as AD.^208,209^ Overexpression of APP mutants or Aβ aggregates increased ER-mitochondria connectivity, resulting in the elevation of mitochondrial calcium.^208,210,211^ C99, a C-terminal fragment of APP cleaved by β-secretase, also activated sphingolipid turnover and increased ceramide to impact the ER-mitochondria contacts, leading to impaired mitochondrial respiration and metabolic disturbance.^212^

Mitochondria are also the major source of oxidative stress because the inevitable leakage of electrons at complex I and complex III of the electron-transport chain to produce reactive oxygen species (ROS).^213,214^ Mitochondria generate approximately 90% of the cellular ROS.^215^ The damaged mitochondria are less efficient to generate ATP but more efficient to produce ROS.^216^ The vulnerability of the brain to ROS is now emerging as a key detrimental factor driving AD pathogenesis. Neurons exposed to ROS stimuli are more susceptible to developing age-related neurodegenerative pathologies, as seen in AD brains. Redox active metal ions, such as Cu and Fe bind to Aβ to produce the ROS, which contributes to the oxidative damage on proteins and lipids leading to impaired membrane integrity, neuronal dysfunction and DNA damage.^217–220^ In addition, mitochondrion-derived ROS modulated the APP processing and triggered Aβ production to form a vicous cycle.^221^

#### Change neurochemical systems

Aβ aggregates interact with glutamatergic neurotransmission, which impairs excitatory synaptic plasticity, leading to cognitive decline.^222–225^ Excessive Aβ peptides induced LTD by inhibiting LTP and making a shift of the NMDAR-dependent signaling cascades.^226^ Thus, Aβ accumulation inhibited the synaptic transmission, resulting in early cognitive impairment.^224^ Aβ-induced LTD is also caused by inhibiting glutamate uptake and stimulating glutamate releasing, which evently elevates glutamate levels in the synapse cleft.^222,225,227,228^ An increase of glutamate activated GluN2B-bearing NMDARs, which further led to calcium-induced LTD and synaptic depression.^85^ Aβ oligomers also regulated the trafficking of NMDARs to change dendritic spine density.^222,227,229^ As with NMDARs, AMPARs are also the principal receptors mediating excitatory synaptic transmission.^230^ It has been identified that APP overexpression and increase of soluble Aβ oligomers are related with the downregulation of GluA1/2 subunits of AMPARs, leading to the inhibition of synaptic plasticity, spine loss, and memory deficits.^231,232^

The basal forebrain cholinergic system is one of the earliest brain regions vulnerable to degeneration during AD.^233^ The correlations between enhanced BACE1 activity, Aβ accumulation with atrophy of basal forebrain and loss of functional connectivity have been found in neuropathological and neuroimaging studies.^234–237^ Furthermore, such an inverse correlation seems to be intensified with the ε4 allele of the apolipoprotein E (APOE) gene, which is one of the strongest risk factors for LOAD.^238^

#### Impair brain networks

Decrease of default-mode network (DMN) functional connectivity has been found in prodromal stages of AD, which is associated with loss of gray matter volume in neocortex and hippocampus.^239,240^ Reduced DMN connectivity only occurs in individuals with elevated baseline Aβ-PET indexes, accelerating cortical atrophy.^241^ Consistent with the findings in humans, aging and AD animal models also show disruptions of functional connectivity in the DMN.^242^ The salience network (SN) identifies salient stimuli and plays an important role in the coordination of the central executive (CEN) and the DMN, whose functional impairment is related to learning and episodic memory deficits in both amnestic mild cognitive impairment (aMCI) and AD.^243^ There is an increased Aβ-PET signal within the CEN and the SN in the progression of AD.^244,245^ A spatial covariance between Aβ aggregates with reduced connectivity and metabolism in the CEN and SN has also been found in AD.^246,247^

### Discovery and development of Aβ-based biomarkers

Based on the amyloid cascade hypothesis, Aβ measurement has been considered as a valuable indicator to assist the diagnosis of AD. In clinical settings, Aβ peptides are most frequently measured in the cerebrospinal fluid (CSF) or through brain imaging of Aβ fibrils with positron emission tomography (PET).^248^ CSF analysis offers a quantitative result of the net effect of Aβ peptides, while.^249,250^ There are four tracers used to detected levels of amyloid in the human brain, including ^11^C-Pittsburgh compound B (^11^C-PiB),^251^ Amyvid^TM^ (flobetapir F18),^252^ Neuraceq^TM^ (florbetaben F18)^253^ and Vizamyl^TM^ (flutemetamol F18).^254^ In practice, reduced concentrations of Aβ_42_ in CSF and increased retention of Aβ tracers in the brain have been considered as early biomarkers of AD.^255–258^ Both biomarkers have been demonstrated to have high diagnostic and prognostic value as they start changing decades before the onset of dementia symptoms.^259–266^ However, CSF- and PET-based measures are not suitable for large-scale screening due to their invasiveness, high cost and low accessibility. Considering the greater availability of blood sampling, blood-based biomarkers become the primary goal in screening for and diagnosing AD in the population and many studies now focus on examining the role of peripheral Aβ and APP in AD development.^267–269^ One such study found that plasma concentrations of soluble β-secretase cleaved n-terminal APP (sAPPβ) were significantly reduced in AD patients compared with age-matched cognitively healthy individuals or patients with behavioral variant frontotemporal dementia (bvFTD), indicating the potential role of sAPPβ as a promising new biomarker of AD.^270^ In addition, there is increasing evidence to support that plasma Aβ acts as an endophenotype of AD, which simultaneously changes with Aβ status in the brain.^271–273^ The blood levels of APP_669-711_/Aβ_42_ and Aβ_40_/Aβ_42_ ratios, as well as peripheral Aβ-bound extracellular vesicles (EVs), have been shown to predict brain Aβ burden.^274,275^ Our group has also identified that circulating Aβ could pass the blood brain barrier (BBB) and enter the brain, contributing to the development of AD.^276^ In contrast, Aβ peptides in the CNS can also move into the circulatory system, where the peptides are phagocytosed by the monocytes or neutrophils, directly degraded by the enzymes, or further transported to the peripheral organs or tissues for degradation or excretion.^28,277^ Recently, the development of single molecular assay (Simoa), an ultra-sensitive immunoassay technology, allows the measurement of Aβ_40_ and Aβ_42_ levels at sub-femtomolar concentration. The availability of reliable and sensitive detection of Aβ peptides in blood makes a promise for early diagnosis and better prognosis of AD.

## The progression of Anti-Aβ therapy

To date, five drugs have been approved for the treatment of AD. Four of these medications are classified as cholinesterase inhibitors (CIs), including tacrine, donepezil, rivastigmine, and galantamine. Most of them are approved to treat Alzheimer’s type in the mild-to-moderate stages, except for donepezil which is administered to patients with severe or late-stage AD. Tacrine has been discontinued in the US due to severe liver toxicity. Unlike these four medications, memantine is an N-methyl-D-aspartate (NMDA) receptor antagonist, which exerts its neuronal protective effects by inhibiting glutamate activity. However, these drugs can only help alleviate the symptoms instead of modifying the disease. Thus, development of effective disease-modifying therapies for AD is urgent and necessary.

According to ALZFORUM (March 2023, www.alzforum.org), 298 AD therapies have been under clinical trials. 76 of them target the Aβ peptide or its aggregates, including small molecules (Table 1) and immunotherapies (Table 2), which can be classified into four categories: (1) to reduce Aβ generation;^278–282^ (2) to enhance the degradation and clearance of Aβ and its aggregates;^283–285^ (3) to neutralize soluble Aβ monomers or its toxicity;^286–294^ (4) to directly inhibit Aβ aggregation.^295–299^ So far, two antibody-based drugs aducanumab and lecanemab have been approved by the FDA and 38 of them have been discontinued due to ineffectiveness or toxic side effects.

**Table 1:** Aβ-related small molecules for AD treatment

Agent	Route	Mechanism of action	Reference
Reduce Aβ generation			
Acitretin	Oral	Increases the expression of α-secretase (ADAM10) to boost the non-amyloidogenic processing of APP and reduce Aβ levels	^278^
Lenalidomide	Oral	Inhibits BACE1 expressions	^280^
Levetiracetam	Oral	N/A	^281^
NIC5-15	Oral	γ-secretase modulator	ALZFORUM
Posiphen	Oral	Blocks the translation of APP	^282^
Enhance the clearance of Aβ or its aggregates			
ALZT-OP1	Oral	Promotes the microglia-mediated phagocytosis of Aβ	ALZFORUM
Bexarotene	Oral	Acts as an agonist of retinoid X receptor to increase brain ApoE concentration	^283^
Destabilize or inhibit Aβ aggregates			
ALZ-801	Oral	Prodrug of the modified amino acid homotaurine that inhibits the aggregation of Aβ_42_ into toxic oligomers by stabilizing Aβ_42_ monomers.	^295^
Contraloid	Oral	Stabilizes Aβ_42_ monomers to inhibit its aggregation	^296^
PBT2	Oral	Lowers extracellular levels of bioactive metals, and thus reduce metal-mediated Aβ aggregation	^298^
Varoglutamstat	Oral	Inhibits the generation of a highly toxic and aggregation-prone form of Aβ (pGlu-Aβ).	^299^
Ameliorate the toxic effects of Aβ aggregates			
ALX-001	Oral	It prevents Aβ-induced synapse loss by competing with metabotropic glutamate receptor type 5 (mGluR5) for binding with Aβ oligomers	^286,287^
CT1812	Oral	Blocks the binding of oligomeric Aβ with its receptors, and thus reduce Aβ-induced synaptic toxicity	^289^
Nasal insulin	Intranasal delivery	Synaptic remodeling and glucose utilization	^290–292^
Simufilam	Oral	Prevents and reverses the binding of Aβ_42_ to α7nAChR, which reduces tau deposition, neuroinflammation and synaptic dysfunction	^293,294^

*Aβ* amyloid β, *AD* Alzheimer’s disease, *APP* amyloid precursor protein, *BACE1* beta site APP cleaving enzyme 1, *CSF* cerebrospinal fluid, *DS* down syndrome, *FDA* Food and Drug Administration, *NfL* neurofilament light, *PET* positron emission tomography

**Table 2:** Immunotherapy targeting Aβ (clinicaltrials.gov accessed March 19, 2023)

Agent	Route	Mechanism of action	Ongoing clinical trials	Clinical outcome	Reference
Targeting Aβ monomers					
ABBV-916	Intravenous infusion	A monoclonal antibody recognizing truncated Aβ modified with pyroglutamate at position 3 (N3), which is aggregated in amyloid plaques	NCT05291234 (Phase 2)	Unpublished	ALZFORUM
ABvac 40	Subcutaneous injection	An active vaccine targeting the C terminus of Aβ_40_	NCT03461276 (Phase 2)	Safe, well tolerated, and consistently elicited a specific immune response in patients with mild to moderate AD	^326^
AV-1959D	Intradermal injection	A DNA vaccine fuses coding sequences of three copies of Aβ1-11 to 12 to elicit antibodies to Aβ peptides	NCT05642429 (Phase 2)	Unpublished	^328^
Aduhelm (Approved by the FDA)	Intravenous infusion	A human IgG1 mAb against a conformational epitope found on the N-terminus of Aβ (residues 3–6)	NCT05310071 (Phase 4)	The highest dose of aducanumab treatment significantly improved cognitive deficit in the participants. In June 2021, aducanumab was approved by the FDA for medical use. As required by the FDA, a Phase 4 confirmatory trial called ENVISION was planned in May 2022. The study will recruit 1500 patients with early AD including participants from black and Hispanic communities in the US	^46–48,347–349^
Donanemab	Intravenous infusion	A humanized IgG1 monoclonal antibody against a pyroglutamate form of Aβ to inhibit its aggregation	NCT05026866 (Phase 3) NCT04437511 (Phase 3) NCT05108922 (Phase 3) NCT04640077 (Phase 3) NCT05508789 (Phase 3)	Slowed cognitive and functional decline as well as reduced plaque loads and tau accumulation in patients with early symptomatic AD but might cause ARIA-E and reduce brain volume. Two Phase 3 trials, including those for prevention and treatment ones are currently underway	^49,344^
MEDI1814	Subcutaneous or intravenous injection	An antibody specific for the C-terminus of Aβ_42_	N/A	Increased CSF Aβ_42_ levels and decreased NfL levels in the plasma. No significant changes in plasma or CSF pTau181, total Tau, or neurogranin were found	ALZFORUM
PRX012	Subcutaneous injection	A humanized monoclonal IgG1 antibody to an N-terminal epitope on Aβ, which stimulates microglia-mediated phagocytosis	N/A	A phase 1 study is ongoing to determine the safety, tolerability, immunogenicity and pharmacokinetics	Press release and company presentation
Remternetug	Subcutaneous or intravenous injection	A monoclonal antibody recognizing truncagted Aβ modified with pyroglutamate at position 3 (N3), which is aggregated in amyloid plaques	NCT04451408 (Phase 1) NCT05463731 (Phase 3)	A phase 1 study is ongoing to determine the safety, tolerability, immunogenicity and pharmacokinetics. A phase 3 trial called TRAILRUNNER-ALZ1 is currently underway. The study plans to recruit 400 patients with early symptomatic AD	ALZFORUM
Solanezumab	Intravenous infusion	A humanized monoclonal IgG1 antibody directed against the mid-domain of the Aβ peptide to reduce Aβ-induced synaptic toxicity	NCT01760005 (Phase 2/3)	Increased in plasma Aβ levels and decreased in CSF Aβ_40_ levels in a dose-dependent way, and may slightly improve cognition in participants with mild but not moderate AD. Phase 2/3 clinical trials are ongoing to assess its effect in participants genetically at risk for early onset AD	^345,346^
UB-311	Intramuscular route	A synthetic peptide vaccine, which neutralizes Aβ toxicity and promotes plaque clearance.	N/A	UB-311 was safe and generated Aβ antibodies in 96% of patients with mild AD. Participants receiving four boosters showed a modest reduction in brain amyloid	^327^
Targeting Aβ aggregates					
ACI-24	Subcutaneous injection	A liposome vaccine designed to elicit an immune response against Aβ aggregates	NCT05462106 (Phase 1 and 2)	Safe, well tolerated, and immunogenic in people with mild AD, but may have no clinical effect as there was no change on amyloid-PET; A Phase 1/2 clinical trial is ongoing to assess its safety, tolerability, immunogenicity, and clinical efficacy in AD in Down’s syndrome (DS) patients	^324^
ACU193	Intravenous infusion	A humanized IgG2 monoclonal antibody to selectively bind with soluble Aβ oligomers	NCT04931459 (Phase 1)	Unpublished	^288^
ALZ-101	Intramuscular injection	Stimulates an immune response specific to soluble Aβ oligomers	NCT05328115 (Phase 1)	A Phase1b study is ongoing	^325^
Crenezumab	Subcutaneous injection or intravenous infusion	Has high affinity with the oligomeric and fibrillar Aβ species, which stimulates the phagocytosis of amyloid plaques	Discontinued	Safe and well tolerated but had no effect on disease biomarkers or clinical decline in participants with prodromal to mild AD. The prevention trial was also negative on the primary outcomes	^339–343^
DNL919	Oral	A TREM2 agonist antibody to stimulate microglia for amyloid phagocytosis	NCT05450549 (Phase 1)	Unpublished	^331^
Gantenerumab	Subcutaneous injection	Human IgG1 antibody designed to bind with a conformational epitope on Aβ fibrils, which recruits microglia to activate phagocytosis	Discontinued	Reduced plaque load and normalized CSF levels of disease biomarkers in AD participants but did not improve cognition (symptomatic) or prevent cognitive decline (asymptomatic). Phase 2/3 clinical trials showed that gantenerumab reduced only half as much as plaque as expected. The results showed the trends of clinical improvement, but fell short of statistical significance	^336–338^
IBC-Ab002	Intravenous infusion	Recruits regulatory T cells and monocytes to stimulate amyloid clearance and alleviate inflammation	NCT05551741 (Phase 1)	A Phase 1, first-in-human study has begun to evaluate the safety, tolerability, pharmacokinetics, and immunogenicity of intravenous IBC-Ab002 in AD patients	^285^
Lecanemab (Approved by the FDA)	Subcutaneous or intravenous injection	A humanized IgG1 version of the mouse mAb158, which specifically binds to large, soluble Aβ protofibrils	NCT03887455 (Phase 3) NCT04468659 (Phase 3) NCT01767311 (Phase 2) NCT05269394 (Phase 2/3)	Reduced brain amyloid and improved cognitive decline in the highest-dose group (twice-monthly 10 mg/kg). The results of the Phase 3 study showed that patients with lecanemab treatment had lower brain amyloid levels and reduced cognitive and functional decline as measured by the Clinical Dementia Rating-Sum of Boxes (CDR-SB), by 27% compared with placebo. Routine MRI scans showed around 21% of individuals on lecanemab experienced side effects such as ARIA, compared with just over 9% in placebo-treated controls	^50,350–353^
Trontinemab	Intravenous infusion	A new version of gantenerumab with Roche’s “brain shuttle” technology to have a better ability of crossing the BBB	NCT04639050 (Phase 1/2)	No safety events were observed in the phase 1 study. Another phase 1 study was begun in March 2021, which includes 120 people with prodromal or mild to moderate AD and a positive amyloid PET scan	https://www.alzforum.org/news/conference-coverage/shuttle-unloads-more-gantenerumab-brain

*Aβ* amyloid β, *AD* Alzheimer’s disease, *ARIA* amyloid-related imaging abnormality, *BBB* blood-brain barrier, *CSF* cerebrospinal fluid, *DS* down syndrome, *FDA* Food and Drug Administration, *MRI* magnetic resonance imaging, *NfL* neurofilament light, *PET* positron emission tomography

### BACE1 inhibitors

In 1999, BACE1 was identified as an enzyme required for Aβ production.^300–303^ Since then, inhibiting BACE1 activity has been pursued as a key method of halting the amyloid cascade and the development of effective BACE1 inhibitors has become a focus of many drug trials. LY2886721 was the first BACE inhibitor to reach Phase 2 clinical trials.^304^ Compared to the previous compound, it has better brain penetrance. In 2012, Eli Lilly announced that the application of LY2886721 produced the expected results in Phase 1 studies with reduced CSF levels of Aβ_40_ and Aβ_42_ as well as increased sAPPα levels (P3-359, Alzheimer’s Association International Conference, 2012). However, it was halted in the Phase 2 study due to abnormal liver biochemistry values in four participants. Its toxicity was considered to be an off-target effect of the compound, which was not related to BACE1 inhibition (The 11^th^ International Conference on Alzheimer’s & Parkinson’s Disease, 2013). Besides LY2886721, many other candidates have also reached late stages of clinical trials, including atabecestat (Phase 2/3),^305^ elenbecestat (Phase 3),^306^ lanabecestat (Phase 2/3)^307,308^ and umibecestat (Phase 2/3).^309^ However, all of them have failed to receive final approval to reach the market. Several obstacles have been found in the development of effective BACE1 inhibitors. BACE1 possesses structural similarities with many other aspartyl proteases, such as BACE2, pepsin, renin, cathepsin D and cathepsin E, a significant challenge to achieve the selectivity in BACE1 inhibition without affecting other proteases that cause off-target side effects.^310^ In addition, the size of the BACE1 active site is relatively large, including catalytic aspartic acid residues, flap, and 10 S loop.^311^ Since all the developed BACE1 inhibitors are small molecules, it may be difficult to occupy this large active site to efficiently block BACE1 activity. Low penetrance of blood-brain barrier (BBB) is also another concern.^312^

### γ-secretase inhibitors/modulators

γ-secretase inhibitors (GSIs) have been widely investigated as potential therapeutic approaches for AD due to their ability to inhibit Aβ production. However, the existing GSIs act too generally, which causes serious side effects through inhibiting the processing of other proteins, such as Notch, a transmembrane receptor involved in regulating cell-fate decisions.^15,313^ Thus, researchers have tried to develop a much more specific γ-secretase inhibitor, which only disrupts the production of Aβ but not others. Avagacestat is a recently developed arylsulfonamide γ-secretase inhibitor with high selectivity for APP over Notch, which successfully reduces CSF Aβ levels in the animal models without any Notch-related toxicity.^314^ Avagacestat was considered as a promising AD treatment with the ability to selectively inhibit the APP processing without affecting the Notch pathway. However, it was terminated in Phase 2 trials due to gastrointestinal and dermatological side effects.^315^ These failures popularized the development of γ-secretase modulators (GSMs) as an alternative approach. GSMs aim to regulate but not totally block the enzyme’s activity. A recent study found that treatment with one potential candidate, SGSM-36, which successfully reduced the level of toxic Aβ_42_ peptides, without changing the proteolytic processing of Notch or α- and β-secretase processing of APP.^316^ EVP-0962 is another GSM that was shown to reduce Aβ_42_ levels and increase Aβ_38_ levels without affecting Notch signaling in vitro. It also improved the memory deficits in AD model mice.^317^ Unfortunately, all of them have been discontinued in the clinical trials.

### Active and passive immunotherapy

Immunotherapy has been considered as one of the most promising strategies aimed at the modification of AD development. This approach involves designing synthetic peptides or monoclonal antibodies (mAbs) to decrease brain Aβ load and slow the disease progression. The first AD vaccine tested in a clinical study was AN1792, a synthetic full-length Aβ_42_ peptide.^318^ Although the vaccine showed some therapeutic effects, including slowed cognitive decline, the clinical trials were terminated due to the occurrence of aseptic meningoencephalitis in 6% of the participants.^319–321^ A possible explanation for this side effect is the induction of T helper 2 (Th2) cell responses by the excipients applied to produce C-terminus region of Aβ peptides.^321^ Accordingly, the subsequent vaccines do not include this region of Aβ peptides. Vanutide cridificar (ACC-001) is a conjugate of multiple short Aβ fragments to avoid the safety concerns associated with AN1792.^322^ Preclinical data showed that vanutide cridificar induced the generation of N-terminal anti-Aβ antibodies and successfully improved cognitive impairment in AD animal models. However, all clinical trials using vanutide cridificar were also discontinued following a serious adverse event.^323^ Another example is Lu AF20513, which is a mixed peptide containing three repeats of the first 12 amino acids of Aβ peptide interspersed with tetanus toxin sequences. The peptide was designed to activate a B cell response to produce polyclonal antibodies against Aβ. While Lu AF20513 was shown to successfully remove brain amyloid deposits in the initial preclinical study, clinical trials were terminated due to a lack of efficacy.^324^ Currently, four vaccines are under the clinical trials, including ALZ-101 (Phase 1), ACI-24 (Phase 2), ABvac 40 (Phase 2) and UB311 (Phase 3). ALZ-101 is a vaccine specific to soluble Aβ oligomers rather than Aβ monomers or fibrils.^325^ It is undergoing a Phase 1B study. ACI-24 is a liposome vaccine based on the Aβ_1-15_ sequences. It is designed to generate antibodies specifically against the β-sheet folding of Aβ. In the preclinical studies, ACI-24 was shown to generate high titers of anti-Aβ IgG1 and IgG2b antibodies and improve novel object recognition in AD mice.^324^ Its Phase 2 trials have been started, in which ACI-24 becomes the first anti-Aβ vaccine to be evaluated for treating AD in Down’s syndrome patients. Another vaccine called ABvac40 targets the C-terminus of Aβ peptides and is also currently being evaluated in Phase 2 clinical studies.^326^ UB-311 consists of the Aβ_1-14_ peptides in combination with a Th-cell epitope, which was designed to specifically stimulate Th2 cells regulatory immune responses over Th1-mediated autoimmune responses. UB-311 was shown to neutralize Aβ toxicity and enhance plaque clearance in preclinical studies.^327^ In the Phase 2 studies, UB-311 also showed its safety and generated Aβ antibodies in 96% of the patients with mild AD (14^th^ International Conference on Alzheimer’s and Parkinson’s Disease, 2019). In 2020, it was announced that UB-311 would begin a Phase 3 clinical testing, in which two double-blind, placebo-controlled studies will be conducted. However, the data related to this clinical trial have not been released. In May 2022, UB-311 was granted fast-track designation by the FDA for Alzheimer’s treatment.

Passive immunotherapy prevents some issues of the active immunization by using monoclonal antibodies (mAbs) directly targeting different forms of Aβ peptides, including monomers, oligomers and fibrils to inhibit the formation of toxic aggregates.^328–331^ The Fc domain of mAbs binds to the Fc-γ receptors on the microglia, leading to the phagocytosis of the Aβ-mAb complex.^332^ In addition, the Aβ-mAb complex induces the complement-dependent cytotoxicity, resulting in the lysis of the target cells. In the blood, the mAbs interact with Aβ to reduce Aβ concentration, resulting in a concentration gradient that stimulates the efflux of Aβ from the brain.^333^ Bapineuzumab is the first antibody to be tested in clinical trials. It is a humanized version of the mouse anti-Aβ monoclonal 3D6 antibody specifically targeting the N-terminal region of Aβ (residues 1–5). Humanized antibodies are generated by modifying protein sequences from non-human species to increase their similarity to natural antibody variants produced in humans, which reduce the immunogenicity of the antibodies, enhance human effector functions, and increase the serum half-life of the antibodies in humans.^330^ In the preclinical studies, 3D6 binds to monomeric, oligomeric and fibril forms of Aβ, leading to the reduced levels of Aβ and improved cognitive deficits in AD model mice.^334^ However, the Phase 3 clinical trials revealed that bapineuzumab could not improve clinical outcomes in mild to moderate AD patients.^335^ There are also other candidates under the Phase 3 trials, including gantenerumab, crenezumab, donanemab, solaneuzumab and lecanemab (BAN2401). Gantenerumab is a human mAb designed to bind with a conformational epitope on Aβ aggregates. It reduces the plaques by stimulating the microglia-mediated phagocytosis. The antibody was found to be safe and well tolerated during the Phase 1 clinical trials, except that transient amyloid-related imaging abnormalities (ARIA) appeared in some patients given a high dosage.^336^ The initial results of phase 2 studies suggested gantenerumab may have no efficacy in the enrolled cohort. However, subsequent post-hoc analyses showed a slight benefit in patients with fast disease progression. It was also tested in a Phase 2/3 study called the Dominantly Inherited Alzheimer Network Trials Unit (DIAN-TU) aimed at preventing dementia in 210 people who were in the progression to Alzheimer’s disease due to an inherited autosomal-dominant mutation in *APP*, *PSEN1*, or *PSEN2*.^337^ Gantenerumab treatment significantly reduced the amyloid loads and normalized CSF Aβ_42_ levels.^338^ However, cognitive data revealed that gantenerumab did not reach its therapeutic point. In addition, two Phase 3 trials were conducted in prodromal or mild AD patients with amyloid deposition. Just several months ago (Nov 14, 2022), Roche and Genentech announced that the outcome of the Phase 3 trials were disappointing, in which the drugs failed to slow cognitive impairment. A new version of ganterumab, called trontinemab is currently under Phase 1 trial, which contains a Fab fragment for better penetration to the BBB. Compared with unmodified ganterumab, 50 folds more trontinemab entered the brain and bound to Aβ plaques. Similar to gantenerumab, crenezumab also recognizes multiple forms of Aβ aggregates. It has high affinity with the oligomeric and fibril species and amyloid plaques.^339,340^ Crenezumab is being tested in both prevention and treatment paradigms.^341–343^ Unfortunately, most of the initial trials including the prevention trial failed to achieve their primary endpoints, and crenezumab is now discontinued. Donanemab is a humanized IgG1 monoclonal antibody targeting the existing amyloid plaques and clearing them from the brain.^344^ Early results from the Phase 1 and 2 clinical studies offered some compelling evidence that donanemab could slow down the amyloid and tau burden. As a result, donanemab has been granted the Breakthrough Therapy designation by the FDA and two Phase 3 trials, including those for prevention and treatment ones, are currently underway. In early of this month (May 3, 2023), Eli Lilly announced partial results of the Phase 3 study showing that donanemab significantly slowed cognitive and functional decline by 35% in patients with early symptomatic AD. In addition, 47% of the participants with donanemab for 1 year showed no clinical progression compared with 29% participants on placebo. The drug achieved its best effect in patients with moderate levels of tau proteins. However, its side effects of bleeding and seizures caused by ARIA also raise big concerns. Solaneuzumab is the humanized version of the murine m266 IgG1 mAbs that target the central region of Aβ. It has more affinity to Aβ monomers than the toxic aggregates. Although solanezumab was well tolerated in the participants, it was not able to show the significant therapeutic benefits to AD patients.^345,346^ The failure may be due to the too low concentrations of the antibody reaching to the brain.

Aducanumab is the first FDA-approved therapy for Alzheimer’s.^47,48^ It is a human IgG1 mAb against a conformational epitope found on the N-terminus of Aβ (residues 3–6), and thus specifically targeting aggregates rather than monomers. It has been shown to reduce plaques in imaging studies.^347^ However, in 2019, Biogen and Eisai announced they would not start an anticipated Phase 3 secondary prevention program and would terminate all ongoing trials as aducanumab treatment was predicted to miss its primary endpoint based on the interim analysis (Mar 2019 news, www.eisai.com). Later, Biogen announced that the interim futility analysis was wrong and the highest dose of aducanumab treatment significantly improved cognitive deficit in the participants (Oct 2019 news, investors.biogen.com). In June 2021, aducanumab was approved by the FDA for medical use.^47,48^ However, it is considered controversial due to the lack of sufficient evidence to support its efficacy.^348,349^ As required by the FDA, a Phase 4 confirmatory trial called ENVISION was planned in May 2022. The study will recruit 1500 patients with early AD including at least 18% of participants from black and Hispanic communities in the US (Jan 2022 news, investors.biogen.com).

Lecanemab (BAN2401) is the humanized IgG1 version of the mouse mAb158, which specifically binds to large, soluble Aβ protofibrils. The antibody has been proved to be safe without serious adverse events in the Phase 1 trials.^350^ In the Phase 2 trial, it had been identified to successfully reduce brain amyloid and improved cognitive decline in the highest-dose group (twice-monthly 10 mg/kg).^351^ A Phase 3 study called Clarity AD was initiated in March 2019 to determine the therapeutic efficacy of lecanemab on 1795 people with mild cognitive impairment (MCI) or early Alzheimer’s disease. The results were just published and showed that patients with lecanemab treatment had lower brain amyloid levels and reduced cognitive and functional decline as measured by the Clinical Dementia Rating-Sum of Boxes (CDR-SB), which quantifies symptom severity across a range of cognitive and function domains, by 27% compared with placebo.^50^ The positive results made lecanemab become another FDA-approved treatment of Alzheimer patients with mild cognitive impairment. However, routine MRI scans showed around 21% of individuals on lecanemab experienced side effects such as ARIA, compared with just over 9% in placebo-treated controls.^352^ ARIA may further cause brain atrophy showing as the increased size of the ventricle. In Feb 2020, it was announced that a large lecanemab study called AHEAD3-45 would run from July 2020 to October 2027 to measure the preventive effect of lecanemab treatment on amyloid and tau tangle formation.^353^

## The stumbling block of anti-Aβ therapy

### Disturbed physiological functions of soluble Aβ

**Fig. 4:**
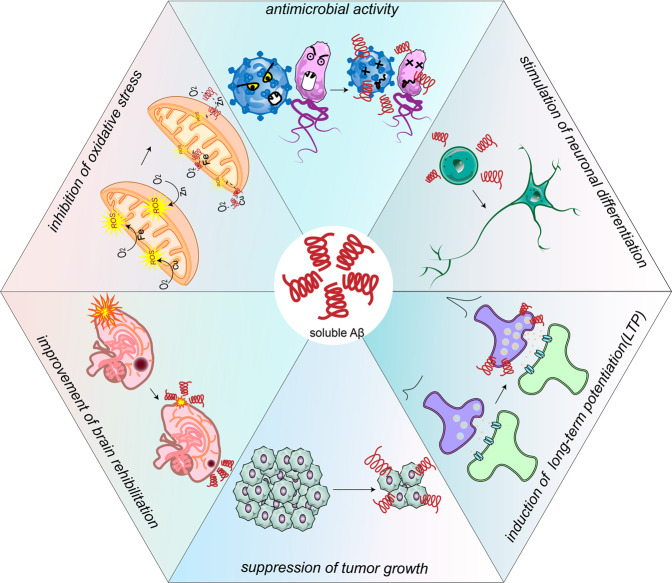
The physiological functions of soluble Aβ. Soluble Aβ at physiological levels has been identified to have some important functions, including induction of long-term potentiation (LTP), stimulation of neuronal differentiation, improvement of brain recover from injuries, inhibition of oxidative stress, antimicrobial activity and tumor suppression

Aβ peptides exist in both the brain and blood throughout an individual’s life.^354^ Although the aggregates have been considered to be toxic, soluble Aβ at physiological levels have been identified to have biological functions, including enhancement of long-term potentiation (LTP),^355–358^ stimulation of neuronal differentiation,^359^ improvement of the brain’s ability to recover from injuries,^360–363^ inhibition of oxidative stress,^364^ antimicrobial activity^365^ and tumor suppression^366,367^ (Fig. 4). These physiological functions must be taken into consideration when strategies are developed to lower Aβ levels in AD. Ideally, such strategies should have more precise targeting of conformations, which are fibrils protofibrils or oligomers, and maintain normal physiological level of Aβ monomers.

#### Modulation of synaptic function

Although Aβ aggregates, especially the soluble oligomeric species impair synaptic plasticity by inhibition of LTP and induction of LTD, growing evidence indicates that a normal level of Aβ peptides may play a key role in the maintenance of synaptic function and cognition.^368,369^ It has been shown that the KLVFF (16~20 amino acid sequence) of Aβ peptides has a protective effect against excitotoxicity, which prevents neuronal death.^370^ In addition, both synthetic and endogenous Aβ_42_ monomers in nanomolar concentrations stimulated the activity of cyclic adenosine monophosphate (cAMP) responsive element-binding protein (CREB) and brain-derived neurotrophic factor (BDNF), which possessed key roles in the regulation of gene expressions related to neuronal functions and survival in normal brains.^371,372^ In contrast, removal of endogenous Aβ by injection of anti-Aβ antibodies or genetic manipulation greatly decreased LTP and impaired memory, which could be rescued by the addition of human Aβ_42_.^357,373–376^ Together, the possible role of Aβ peptide in the modulation of synaptic function as well as learning and memory has been suggested. Aβ monomers stimulated astrocytes to increase the clearance of synaptic glutamate and therefore protect neurons from glutamate excitotoxicity.^377,378^ Aβ can also be released into the synaptic cleft, where it acts on presynaptic neurons to induce the release of neurotransmitters (e.g. acetylcholine) or directly activates α7-nicotinic acetylcholine receptors (α7-nAChRs) to enhance long-term potentiation (LTP).^355–358^ In the CNS, the nicotinic acetylcholine receptors (nAChRs) are expressed in both neurons and non-neuronal cells.^379,380^ As ligand-gated ion channels, nAChRs opened in response to the depolarization of the membrane, allowing Na^+^, K^+^ and Ca^2+^ to enter the cells.^381,382^ Among the isofroms, the α7-nAChRs had the highest Ca^2+^ permeability.^381^ The mechanism behind Aβ-induced α7-nAChR activation could be due to the disruption of intracellular signal transduction to stimulate the calcium influx.^383^ α7-nAChRs are involved in a variety of biological functions, including neurotransmitter release, synaptic plasticity and neurogenesis.^384,385^ In AD brain, nAChRs have been detected in Aβ_42_-positive neurons and their reduction is associated with disease progression.^386^ Furthermore, there was an increase of Aβ/nAChR-like complexes in carriers of APOE ε4, a strong risk factor for LOAD.^387^ In fact, Aβ might interact with specific subtypes of nAChRs with different structures to mediate its physiological effects or toxicity to cholinergic neurons. Under physiological conditions, low level of Aβ particularly interacted with the α7 isoform via the nitric oxide/cGMP/protein kinase G pathway to activate the channels.^388,389^ Thus, α7-nAChR KO mice at 12-month-old showed Aβ elevation as a compensatory response of α7-nAChRs and exhibited AD-like pathologies.^390^

Inhibition of APP changed the expressions of post-synaptic proteins such as GluA1subunit of AMPA receptors, suggesting the involvement of APP in synaptic formation.^391^ An obvious reduction of LTP was found in cultured hippocampal neurons with knockdown of APP expression.^392^ Similarly, conditional KO of *PSEN1* and *PSEN*2 to inhibit Aβ production also led to impaired synaptic plasticity and cognitive deficits in animal models.^393^ In contrast, application of nanomolar synthetic Aβ successfully enhanced the cognitive and memory performance of the mice.^357^ However, the nanomolar concentrations of Aβ used in the study deviate too far from the physiological level of Aβ in picomolar concentrations. To address this concern, other studies injected picomolar concentrations of Aβ peptides into the mice, which also significantly enhanced synaptic plasticity and memory formation.^394^ These findings suggest that physiological levels of Aβ monomers are crucial to maintain a normal synaptic function while only Aβ aggregates have the inhibitory and toxic effects.

#### Promotion of injury recovery

Evidence from patients and animal models also shows rapidly increased Aβ expressions after being injured are beneficial,^360–363^ indicating the role of Aβ in stimulating the brain to recover from traumatic and ischemic injuries. There is an elevation of Aβ peptides during traumatic brain injury (TBI), indicating that Aβ may belong to the pathological cascade of TBI or be an agent for improving recovery.^395,396^ To answer this question, Aβ_40_ peptides were intracerebroventricularly injected into TBI-impacted BACE1^–/–^ mice, which significantly improved motor memory deficits in these injured mice, suggesting the protective effect of Aβ.^362^ In contrast, reduction of endogenous Aβ levels by using γ-secretase inhibitor DAPT or deleting the enzyme BACE1 attenuated the functional recovery in mice with spinal cord injury (SCI).^396^ Aside from TBI, Aβ may also have a protective role against other types of brain injury such as cerebral ischemia, which blocks the blood flow in brain. It has been demonstrated that overexpression of human APP (hAPP695) leads to an obvious lower infarct volume in the cortex of mice suffering from cerebral ischemia.^363^ Experimental autoimmune encephalomyelitis (EAE) is a T cell-mediated autoimmune disease with inflammation in brain. Aβ treatment was found to effectively inhibit the production of proinflammatory T helper cells (TH1 and TH17) and the related cytokines including IL-6, IFN-γ and IL-17, which improved motor paralysis in EAE animal models. In constrast, genetic deletion of *APP* significantly aggravated the severity of the disease, suggesting the protective role of Aβ against autoimmune inflammation in CNS.^397^

#### Anti-microbial activity

Recently, Aβ’s role as an anti-microbial peptide has been demonstrated. Animal models with the expression of human Aβ showed stronger resistance to bacterial and viral infections.^365^ Moreover, brain tissues from AD patients show higher anti-microbial activity than samples from age-matched non-AD individuals, which was correlated with Aβ levels in brain.^398^ It is hypothesized that the anti-microbial activity of Aβ is associated with its capacity to bind with microorganisms and form a net to trap the infectious agents.^399^ This idea fits with the findings that HSV1 and Borrelia DNA have been found in plaque cores of AD brains.^400,401^ Aβ peptides are able to interact and entrap various bacterial strains and viruses, such as HSV1 and HSV6, block their entry into the host cells to replicate.^402–404^ Interestly, Aβ_42_ cannot prevent the replication of non-enveloped human adenovirus, suggesting that it probably interacts with viral coat proteins.^404^ Aβ stimulated the aggregation of viral particles, which facilitated leukocyte-mediated uptake of viruses.^405^ In addition, the damaged host cells released nucleic acids containing Aβ aggregates, which were immunogenic and elicited the secretion of type I interferons (IFNs) by adjacent microglia to accomplish the antiviral response.^406^ The produced interferon-γ (IFN-γ) further facilitated Aβ generation to form a positive feedback loop.^407^ Similar to anti-viral activity, Aβ peptides also bound to fungal cells and stimulated the phagocytosis of microglia.^408^ Thus, familial AD mutations accelerated the clearance of C. albicans from brains in mice.^408^ Together, the underlying mechanisms of Aβ peptides exerting their anti-microbial activity including interation with membranes and disruption of membrane integrity; stimulation of phagocytosis by inducing cytokines or altering microorganisms’ conformation.

#### Suppression of tumor growth

In addition, recent studies show that AD patients have significantly lower incidences of several types of cancers, including skin cancer, lung cancer, breast cancer and bladder cancer.^366,367^ Aβ has been demonstrated to inhibit tumor cell growth. In vitro, application of media containing Aβ successfully inhibits the proliferation of cells, including human glioblastoma, human breast adenocarcinoma, and mouse melanoma cells.^409^ In vivo, injection of Aβ into mice transplanted with human glioblastoma and lung adenocarcinoma suppresses the tumor growth.^410^ In transgenic mice with the expression of human Aβ, the growth rates of implanted glioma tumor masses are inhibited by 40–50% compared to tumor masses in age-matched wild-type mice.^411^

A hypothesis has been proposed that Aβ may promote apoptosis, which contributes to its anti-tumor effects. Aβ_42_ peptides enhanced the transcription of p53, which is responsible for controlling cell apoptosis.^412,413^ In addition, Aβ_42_ induced oxidative stress and decreased the expression of X-linked inhibitor of apoptosis (XIAP), which directly inhibited key proteases of the apoptosis pathway including caspase 3, 7 and 9.^414,415^ Bcl-2, another key anti-apoptotic protein, was also shown to be blocked by Aβ_42_ peptides.^416^ In contrast, Aβ_42_ stimulated the expression of Bax, which induced cell apoptosis and was commonly observed in many cancers.^416,417^

#### Inhibition of oxidative stress

A large amount of studies have shown the anti-oxidant properties of Aβ peptides.^418–420^ Both Aβ_40_ and Aβ_42_ in physiological concentrations prevented lipoprotein oxidation in CSF and plasma.^364,421^ In addition, the increased generation of Aβ by cells from Alzheimer’s patients with mutant *PSEN1* was accompanied by a reduction of ROS levels.^422^ Conversely, application of Aβ to primary hippocampal neurons from *PSEN1* mutant knock-in mice significantly increased superoxide production.^423^ Physiological amounts (picomolar concentrations) of Aβ peptides could function as anti-oxidants by inhibiting redox metals, such as Cu, Fe and Zn to bind with ligands in redox cycling.^364^ The absence of Aβ in neurons may inhibit adequate chelation of metal ions and appropriate removal of O2^-^, resulting in an increased rather than a reduced oxidative stress.^424^ Thus, the physiological anti-oxidant activity of Aβ peptides should be taken into account when designing therapeutic drugs to lower Aβ levels.

#### Stimulation of neurogenesis

Adult neurogenesis in humans was first reported in 1998, in which bromodeoxyuridine (BrdU)-positive cells were found in the post-mortem brain tissue of cancer patients.^425^ Adult brains contain resident neural stem/progenitor cells (NSPCs), which have multipotency and show great potential for self-renewal.^426,427^ Adult neurogenesis in AD brains was also widely investigated. Compared with brain tissues from non-demented individuals, AD brains had increased expressions of DCX, PSA-NCAM, TOAD-64/Ulip/CRMP (TUC-4) and NeuroD, indicating the enhanced neurogenesis.^428^ However, some contradictory results have also been reported. It has been demonstrated that the expression of microtubule-associated protein (MAP) isoforms MAP2a, a marker of the mature neuron, was dramatically decreased in the dentate gyrus of human AD brains, indicating a reduction of neuronal maturation in the hippocampus.^429^ Another study also found a reduced number of DCX- and Sox2-positive cells in the AD hippocampus as compared with non-demented controls.^430^ Furthermore, a study including 45 Alzheimer’s patients between 52 and 97 years of age identified that the number of DCX-positive cells declined with the neuropathological progression.^431^ Growing evidence has shown the effects of Aβ on neurogenic process using NSPCs.^359,432^ Both Aβ_40_ and Aβ_42_ peptides have been identified to induce the proliferation and differentiation of neural progenitor cells (NPCs).^359,432^ Aβ_40_ mainly drived differentiation of NPCs into neurons, differing from Aβ_42,_ which increased glia markers in NPCs.^359^ It has been identified that Aβ peptides stimulate neurogenesis in the subventricular zone (SVZ) through interacting with the p75 neurotrophin receptors in adult mice.^433^

#### Maintenance of BBB integrity

The blood-brain barrier (BBB) contributes to a stable brain microenvironment and normal neuronal function. Although neurotoxic Aβ aggregates play a key pathological role in the damage of the BBB, a low level of Aβ peptides may act as a seal to maintain the integrity of the BBB.^434^ This hypothesis is supported by the role of Aβ as a metal chelating antioxidant to maintain structural integrity under stress conditions.^435^ The ability of binding with copper ion or extracellular matrix molecules allows Aβ with its small size to be an excellent candidate molecule, which could form a “scab” in the brain. Thus, a rapid generation and deposition of Aβ in stroke and after head trauma, which could benefit to maintain the BBB integrity and inhibit the leakage of serum components into the brain, leading to neuroinflammation.^436^

### Insufficient specificity

γ-secretase has dozens of substrates. Previous clinical trials of γ-secretase inhibitors have failed, in large part due to the toxicity induced by lack of substrate-specific inhibition. Particularly notable is toxicity resulting from inhibition of Notch-1 cleavage, which disrupts essential signaling from this receptor.^15,313^ Thus, we should discover compounds that act as substrate-selective γ-secretase inhibitors, which block the cleavage of C99, the immediate precursor of Aβ, while allowing Notch cleavage to proceed unimpeded. Recently, a study showed that verteporfin only bound with the APP transmembrane domain rather than the transmembrane domain of the Notch-1 receptor, indicating its inhibitory effect is in a C99-specific manner.^437^ Our study also showed that *PSEN1*_*S*_*169del* (a deletion mutation in *PSEN1* gene exon 6) has distinct effects on APP processing and Notch1 cleavage.^39^ This AD pathogenic mutation altered APP processing and Aβ generation without affecting Notch-1 cleavage and Notch signaling in vitro and in vivo. The results indicate that serine169 in PS1 could be a critical site as a potential target for the development of novel γ-secretase modulators without affecting Notch-1 cleavage to treat AD.

A lack of selectivity is also a significant barrier to the therapeutic application of BACE1 inhibitors in AD. For instance, BACE2 is a close homolog of BACE1 but plays a neuroprotective role by inhibiting the amyloidogenic pathway of APP processing^7,8,10^ and reducing potassium channel Kv2.1-induced neuronal apoptosis.^438^ Thus, a non-selective BACE1 inhibitor also inhibits BACE2’s protective functions, leading to off target side effects. Although the aspartyl protease family (e.g. BACE2, pepsin, renin, cathepsin D and cathepsin E) has conserved catalytic aspartic acid residues, the subsites in the active sites may be unique.^439^ Targeting these subsites to develop BACE1 inhibitors may increase their specificity. Aβ-targeting antibodies also show off-target effects. A recent study identified that antibodies with Fc fragment reduced Aβ burden but also induced the engulgment of neuronal synapses by activating complement receptor 3 (CR3) or Fcγ receptor IIB (FcγRIIB), which exacerbates cognitive impairment in AD mice.^440^

### Lack of accurate animal models

AD can be classified into a genetic and sporadic form of the disease.^441^ More than 99% of AD cases occur at an age >60 years in a sporadic manner, potentiated by various risk factors related to lifestyle.^442^ Less than 1% of all AD cases are early-onset with symptoms developed at an age of 50 s and earlier, and caused by gene mutations in *APP*, *PSEN1* or *PSEN2*.^7,38,39^ In order to study Alzheimer’s pathogenesis and therapeutic strategies, better animal models to recapitulate the natural process of the disease are required.^443,444^ Many transgenic mouse models have been developed and commonly used, including the mice containing mutations in the *APP* (e.g. Tg2576,^445^ APP SweDI,^446^ APP23,^447^ J20^448^ and TgCRND8^449^ mice), *PSEN1* (e.g. PS1A246E,^450^ PS1M146L^451^), *PSEN2* (PS2N141I^452,453^ mice) or combinations (e.g. APP23xPS1-R278I,^454^ APP/PS1,^455^ APPSwe/PSEN1dE9,^456,457^ APP23/PS45 (APPSwe/PS1G384A),^119,458,459^ 5xFAD (APP SwFILon, PSEN1 M146L, L286V)^460^ and ARTE10^461^ mice). Although the human tau gene *MAPT* mutations per se only cause frontotemporal dementia (FTD) rather than AD,^462^ tau mediates Aβ toxicity to promote the pathological process of AD.^92,137^ The interaction between Aβ and tau is under investigation by the generation of transgenic mouse models expressing human tau and APP, including APP/PS1/rTg21221,^463^ 3xTg-AD (APP Swedish, MAPT P301L and PSEN1 M146V)^464^ and PLB1-triple^465^ mice. To avoid the “random integration” problem occurring in the transgenic mice, knock-in mice are generated in place to precisely target a specific locus. AD knock-in/out mice have been employed, including *APP* knock-in/out,^466,467^ *APP*^NL-F^ knock-in,^468^ *APP*^NL-G-F^ knock-in^468^ and *APP*^NL-G-F^/MAPT double knock-in^469,470^ mice. However, such mouse models only mimic the familial AD with an early onset of the disease. The late-onset sporadic AD is induced by a combination of genetic (e.g. *Apolipoprotein E4* and *TREM-2*),^101,102^ lifestyle and environmental factors.^471–473^ Unfortunately, the current animal models are unable to exactly reflect this complexity, such as aging, which is the major risk factor of sporadic AD. The immune system has long been implicated as an important factor in Alzheimer’s development.^474^ However, murine immune system is notably different from humans.^475^ Furthermore, the extensive neuronal loss in AD patients has not been replicated in the murine models.^476^ Thus, a lack of accurate disease models leads to a translational gap between animal research and the clinical setting. Design and exploration of patient-based research models will be required, which will be further discussed in Section “Perspective and Future Direction”.

### Late application

PET imaging allows us to visualize Aβ fibrils in patients, which accumulate in an Alzheimer’s brain as early as 15 years before the onset of symptoms.^477^ A change in CSF Aβ levels can be detected even up to 25 years before a patient begins to show symptoms.^478^ Thus, the current application of Aβ therapies may be too late for symptomatic patients, whose therapeutic window has already closed. Compared with curing the disease, prevention by reducing the risk of Alzheimer’s development is believed to be more practical. Prevention trials stand a chance to prevent or slow the progression of cognitive decline and dementia in AD. In 2012, DIAN-TU launched the first prevention trial focusing on two drugs: gantenerumab (against Aβ aggregates) and solanezumab (against soluble Aβ monomers).^337^ The data showed that gantenerumab had a positive impact on the reduction of cortical amyloid, leading to its further study by an exploratory open-label extension (OLE).^338^ Crenezumab is the first immunotherapy to be evaluated in the Alzheimer’s Prevention Initiative.^343^ The participants in this trial were carriers of the autosomal-dominant gene mutation (e.g. PSEN1 E280A) but did not meet the criteria for mild cognitive impairment at the time of enrollment.^341^ Although crenezumab did not significantly improve cognitive impairment in the participants, it showed some favorable effects (Alzheimer’s Association International Conference, 2022). Discovery of new biomarkers to discriminate the very early stage of sporadic AD is essential for the success of AD prevention.

## Perspective and future direction

Although the failed trials have fueled debate on the amyloid hypothesis and raised concerns as to if efforts have been properly directed, it has provided valuable lessons to learn from and information that may improve our understanding of Alzheimer’s pathogenesis and drug development. The following are some principle and practical approaches we believe could be beneficial for future Aβ-targeted drug development and therapy.

### Combination therapy and mechanism-based therapy

Some current therapeutic approaches, such as BACE inhibitors and γ-secretase inhibitors/modulators, aim to target Aβ production, which is the early stage of the amyloid cascade.^304–306^ Although these inhibitors have been identified to slow down the plaque formation in patients, they were unable to clear the existing Aβ plaques and ameliorate toxic events already initiated by these Aβ aggregates. Accordingly, combination therapy should be considered for the clinical phase of the disease, which is already the standard of care for many diseases, including rheumatoid arthritis and HIV/AIDS.^479,480^ Growing evidence indicates that Aβ accumulation stimulates tau phosphorylation and fibrillary tangle formation, leading to the process of neurodegeneration.^112–114^ Thus, additional application of tau-phosphorylating kinase inhibitors or compounds that inhibit tau aggregation and/or promote aggregate disassembly should be beneficial. APP and Aβ can be imported into mitochondria, where they can interact with mitochondrial components, impair ATP production, and increase oxidative damage.^481,482^ Antioxidants such as lipoic acid,^483^ vitamin E,^484,485^ vitamin C^486^ and β-carotene^487^ may also be the promising combination approaches for AD. In addition, Aβ’s role in the modulation of synapse function has attracted great attention. The neurotoxic soluble Aβ oligomers have been identified to affect synaptic plasticity and synaptic transmission in various AD animal models.^488^ Targeting synapse loss and dysfunction may be an effective AD treatment strategy.^489^ Once the pathological cascade has begun, combination therapy targeting multiple AD pathologies will be more effective than a single therapy, which only addresses one abnormal factor.

Growing evidence shows that elevation of brain Aβ levels in AD could be the consequence of upstream problems including neurovascular dysfunction, disturbed glucose homeostasis, failed control of cell cycle and inflammation.^490–492^ Autophagy, a part of the lysosomal system, is crucial for clearance of toxic accumulated proteins and damage organelles. The autophagic process consists of several steps including sequestration, elongation, maturation, fusion and degradation, aiming to deliver unwanted proteins, organelles and cellular debris to the lysosome for degration. It starts with the formation of phagophore, which then elongates and encloses the cargo to form an autophagosome. The autophagosome either directly fuses with the lysosome form an autolysosome or firstly fuses with late endosomes to form amphisomes, which subsequently fuse with lysosomes. Impairment of the autophagy-lysosomal system has been considered as one of the fundamental causes for many neurodegenerative diseases that feature the deposition of toxic amyloid proteins. Growing evidence shows that dysfunction of autophagy is closely linked with Aβ metabolism and accmulation in AD progression. Autophagy is implicated in Aβ metabolism likely via modulation of its production, secretion and clearance. Aβ originates from the cleavage of its precursor protein APP by β-secretase (BACE1) and γ-secretase. It has been identified that ATG5-dependent autophagy regulates APP degradation.^493^ In addition, the complex of APP and γ-secretases was found in autophagosomes, suggesting the role of authophgic pathway in the generation of Aβ peptides.^494^ Autophagy is also required for Aβ secretion. ATG7 is an essential molecule for the autophagosome formation. AD model mice with ATG7 KO showed deficient autophagy associated with drastically reduced extracellular Aβ plaques and markedly accumulated intraneuronal Aβ, suggesting that Aβ secretion was compromised due to the impaired autophagy.^495,496^ In addition, autophagy regulates the clearance of Aβ peptides. The cysteine protease cathepsin B (CatB) is a key lysosomal protease required for degrading autophagic substrates. It has been demonstrated that genetic deletion of CatB significantly increased Aβ_42_ burden and worsened amyloid deposition in AD mice, whereas overexpression of CatB reduced amyloid plaques.^497^ Accumulation of immature autophagosome in dystrophic neurites has been observed in the brain of Alzheimer’s patients due to the defective axonal transportation of autophagosomes.^498^ Thus, autophagy modulation becomes a promising stategy for Alzheimer’s treatment.^499,500^ Rapamycin is a commonly used autophagy activator, which inhibits the mTOR pathway by binding with immunophilin FK506-binding protein (FKBP12).^501^ Recent studies identified that 3xTg-AD mice had enhanced mTOR activity in the hippocampus and neocortex, two areas known to have high concentrations of Aβ plaques.^502^ Treatment with rapamycin significantly stimulated autophagy associated with markedly reduced both intracellular Aβ and extracellular amyloid deposition in brains as well as improved cognitive deficits in AD mice.^503,504^

Mechanism-based therapies to target these pathological processes will have optimal benefit when initiated in the asymptomatic stage. Traditional Chinese medicine (TCM) has been established in the Chinese health care system for thousands of years. Most TCM treatment are derived from natural products with multi-target, multi-pathway capacity and mild adverse events. It has preventive and therapeutic effects on many chronic diseases such as cancer, allergy, diabetes and infections by the regulation of cell growth and differentiation, reduction of inflammation, or increase of carbohydrate utilization.^505–508^ TCM treatment such as morroniside, rutin, resveratrol, triptolide and berberine have already shown their beneficial effects for AD^509–527^ (Table 3).

**Table 3:** Multi-target traditional Chinese medicine for Alzheimer’s modification

Agent	Mechanism of action	Reference
Berberine	Activates the PI3K/Akt/GSK3 pathway to reduce Aβ generation; Inhibits the ER stress by blocking the PERK/eIF2α signaling pathway	^509,510^
*Gardenia jasminoides* J.Ellis	Protects the neurovascular unit (NVU) and inhibits the neuroinflammation; Decreases Aβ levels by inhibiting Aβ production and accelerating Aβ degradation	^511,512^
Icariin	Modulates the differentiation of Th1, Th17 and Tregs cells; Inhibits the ER stress by blocking the PERK/eIF2α signaling pathway	^513,514^
*Lonicera japonica* Thunb	Inhibits Aβ aggregation and the subsequent cytotoxicity; Promotes neuritogenesis	^515,516^
Morroniside	Reduces the oxidative stress and tau phosphorylation	^517^
*Platycodon grandiflorum*	Inhibits the oxidative stress by upregulating the antioxidant enzymes; Increases the expressions of Bcl-2 family proteins to inhibit apoptosis	^518,519^
Resveratrol	Reduces Aβ generation by inhibiting the activity of β- and γ-secretases; Stimulates Aβ clearance by activating ADEs and increasing the permeability of the BBB; Increases the levels of estradiol and neprilysin	^520,521^
Rutin	Recruits microglia to promote Aβ clearance; Inhibits the activity of β-secretase and Aβ-induced neuronal depolarization; Reduces the neuroinflammation by downregulating the proinflammatory cytokines	^522–524^
Tanshinone	Reduces the ER stress by blocking the PERK/eIF2α, IRE1α/XBP1 and ATF6 pathways; Inhibits the CHOP or JNK pathways to reduce apoptosis; Inhibits the neuroinflammation by the downregulation of the RAGE/NF-κB signaling pathways	^525,526^

*Aβ* amyloid β, *BBB* blood-brain barrier, *ER* endoplasmic reticulum

### Patient-based research models

Three-dimensional brain organoids derived from human pluripotent stem cells (hPSCs) have shown significant advantages in modeling neurological disorders including autism, microcephaly and Parkinson’s disease.^528–530^ Three methods have been established to recapitulate Alzheimer’s phenotype in brain organoids: application of Aftin-5 (an Aβ_42_ agonist) to induce Aβ_42_ production in brain organoids;^531^ generation of brain organoids from induced pluripotent stem cells (iPSCs) of familial AD patients;^532,533^ and creation of differentiated sporadic Alzheimer’s brain organoids by converting APOE3 to APOE4 in patient-derived iPSCs.^534^ Unlike cell models, AD brain organoids are capable of generating the blood-brain barrier (BBB) as well as connections with other organs.^535^ This enables them to potentially function as a superior approach in the understanding Alzheimer’s pathogenesis, as well as a better tool for exploration of Alzheimer’s modification. In addition, transplantation of brain organoids may be a novel way to recover neuronal function and neural network after neuronal death during AD.^536^ However, some limitations still exist and will need to be improved upon. So far, brain organoids can only be cultured within six months, otherwise volume shrinkage and cellular apoptosis occur as neither the oxygen nor nutrients will be able to reach the innermost organoid regions. This limitation leads to the concern that brain organoids are unable to grow “old” enough to mimic the aging human brain. To address this issue, obtaining brain organoids with a vascular system becomes a critical issue.^537,538^

### Identification of early Alzheimer’s biomarkers

Biomarkers that can identify patients at very early stages of AD will greatly benefit the development of disease-modifying therapies.^539^ In addition to the typical pathologies (e.g. Aβ and tau), other molecules associated with inflammation, synaptic plasticity, may also serve as the accurate and specific biomarkers for early diagnosing AD.^540–542^ Progranulin is a growth factor expressed in neurons and microglia, which modulates neuroinflammatory to reduce microgliosis and astrogliosis.^543^ It has been observed that the CSF level of progranulin elevates as early as ten years before the presentation of symptoms in patients with familial or sporadic AD.^544^ Neurogranin is expressed in the cortex and hippocampus, the brain areas most affected by AD.^545^ As a synaptic marker, it is involved in the modulation of synaptic strength and plasticity.^546^ Several studies have revealed an elevation of CSF neurogranin in AD and MCI individuals compared to healthy controls.^547,548^ The CSF neurogranin levels correlated with the brain amyloid load in patients with preclinical AD. It can also successfully predict the rates of cognitive decline in both early Alzheimer’s patients and cognitively healthy controls. In contrast, there is a significant reduction of plasma neuronal-derived exosomal neurogranin in AD patients compared with the healthy controls.^549^ More importantly, the CSF neurogranin increases exclusively in AD patients and has not been observed in other neurodegenerative disorders, such as frontotemporal dementia or Parkinson’s disease.^550^

MicroRNA (miRNA) are noncoding RNA molecules of 20–25 nucleotides that can manipulate gene expression post-transcriptionally by binding to the 3ʹ-untranslated region (3ʹUTR) of mRNA to block protein translation or accelerating the degradation of target mRNAs. It has been found that miRNAs are involved in Alzheimer’s pathogenesis and are easily detected in body fluids, including CSF, plasma and serum. Therefore, they become an attractive target for developing AD biomarkers.^551,552^ In addition to body fluids, ocular markers also gain increasing interest. Abundant evidence from animal and clinical studies shows a correlation between ocular pathology and AD development.^553–555^ A recent study also suggests that depressive symptoms in middle-aged individuals correlates with time to onset of cognitive decline, suggesting the role of psychiatric disorders as early markers of Alzheimer’s disease.^556^

## Conclusions

Since Aβ aggregates act as the unique specific pathological hallmark of AD and play a causative role in the disease development, they are believed as a promising target for Alzheimer’s modification. Most Aβ-targeting drug trials have failed as a consequence by lack of sufficient specificity and accurate translational models, loss of Aβ physiological homeostasis, and failure to be administered during the best therapeutic window. Nevertheless, learning from these failures will be beneficial to the design of better therapeutic approaches. Biomarkers are needed for identifying patients with preclinical Alzheimer’s disease so that treatment such as mechanism-based therapy could prevent or slow down the disease. Translational models and tools to mimic the nature of AD more closely are also required to bridge the gap between basic research and the clinical practice. Combination therapy that targets different mechanisms and pathologies would be directed by biomarkers and customized to the individual. We hope that these solutions could pave the way for exploration and development of more refined Aβ-based therapy for AD.
